# Optimal control of malaria: combining vector interventions and drug therapies

**DOI:** 10.1186/s12936-018-2321-6

**Published:** 2018-04-24

**Authors:** Doran Khamis, Claire El Mouden, Klodeta Kura, Michael B. Bonsall

**Affiliations:** Mathematical Ecology Research Group, Department of Zoology, University of Oxford, South Parks Road, Oxford, OX1 3PS UK

**Keywords:** Vector control, Optimal control, Cost-effectiveness, Malaria management, Artemisinin, ACT

## Abstract

**Background:**

The sterile insect technique and transgenic equivalents are considered promising tools for controlling vector-borne disease in an age of increasing insecticide and drug-resistance. Combining vector interventions with artemisinin-based therapies may achieve the twin goals of suppressing malaria endemicity while managing artemisinin resistance. While the cost-effectiveness of these controls has been investigated independently, their combined usage has not been dynamically optimized in response to ecological and epidemiological processes.

**Results:**

An optimal control framework based on coupled models of mosquito population dynamics and malaria epidemiology is used to investigate the cost-effectiveness of combining vector control with drug therapies in homogeneous environments with and without vector migration. The costs of endemic malaria are weighed against the costs of administering artemisinin therapies and releasing modified mosquitoes using various cost structures. Larval density dependence is shown to reduce the cost-effectiveness of conventional sterile insect releases compared with transgenic mosquitoes with a late-acting lethal gene. Using drug treatments can reduce the critical vector control release ratio necessary to cause disease fadeout.

**Conclusions:**

Combining vector control and drug therapies is the most effective and efficient use of resources, and using optimized implementation strategies can substantially reduce costs.

## Background

Vector-borne diseases inflict significant levels of human morbidity and mortality. Current estimates suggest that vector borne disease account for 17% of the global disease burden and over half of the world’s population are at risk of contracting a vector-borne disease. Just under half the world’s population live at risk of dengue [1] with more than 300M people contracting dengue annually [1]. Similarly, while the global incidence of malaria is falling, in 2015,  200M malaria infections were reported leading to an estimated 429,000 deaths mostly among African children under the age of 5 [2].

The World Health Organization (WHO) recommends insecticide-treated bed nets (ITNs), indoor residual spraying (IRS) and anti-malarial drug therapies, specifically, the treatment of clinical malaria with artemisinin-based combination therapy (ACT), as the principal methods used to combat malaria [2, 3]. Catalyzed by the Roll Back Malaria Initiative around the United National Millennium Development Goals, a widespread scale-up of coverage of these control interventions successfully reduced and locally eliminated malaria in sub-Saharan Africa; between 2000 and 2015, *Plasmodium* infection in endemic regions of Africa halved and the incidence of clinical disease fell by 40% [4]. This remarkable and widespread reduction is estimated to have averted 663 million clinical cases of malaria since 2000 [4]. However, these gains are fragile and they are increasingly threatened by the emergence of *Plasmodium* strains that are resistant to anti-malarial drugs and mosquitoes that are resistant to the insecticides used to kill them.

Due to widespread resistance to anti-malarial drugs, the WHO recommends ACT as the front-line treatment in all countries with endemic malaria. ACT was first introduced in the mid-1990s and are generally cost-effective [5]. Artemisinin is a highly potent plant-based compound which clears parasitaemia more rapidly than all other currently available anti-malarial drugs [6, 7]. Moreover, genetic mutations which are thought to confer resistance to the anti-malarial drug chloroquine also increase susceptibility to artemisinin and quinine [8]. Therefore, ACT can help delay the development of resistance, as they combine fast-acting artemisinin with another class of anti-malarial drug (such as quinines or anti-folates). Despite these efforts, artemisinin resistance first emerged in the mid-2000s in Cambodia, resulting in longer parasite-clearance times and rising failure rates for some artemisinin-based drug combinations [9]. This resistance has now emerged in or spread across mainland Southeast Asia, appearing in Thailand, Cambodia, Myanmar and Vietnam [10–12]. For now, ACT is still an effective treatment in these countries, as parasites remain susceptible to some partner drugs [13]. However, it is only a matter of time before these drugs begin to fail and while new anti-malarial drugs are being developed, it will be several years before they become available.

By far the most cost-effective intervention for reducing *Plasmodium* infection rates since 2000 has been the widespread roll-out of ITNs—bed nets treated with pyrethroid insecticides [4, 14]. As ITNs kill or disable the mosquitoes which land on them, even modest adoption rates of ITNs can reduce the malaria vector population and achieve community-wide benefits [15]. Unfortunately, although not surprisingly, the intense exposure to insecticides due to the adoption of ITNs and increase in IRS programmes is driving the spread of insecticide resistance in mosquitoes. Pyrethroid resistance was first detected in the mid-1990s [16], and is now ubiquitous in all African malaria vectors and is increasing in strength, meaning mosquitoes can tolerate ever-higher levels of chemical exposure [17]. Several countries have identified mosquito populations that are starting to develop resistance to all four classes of insecticide that are approved by the WHO for IRS (organochlorines, organophosphates, carbamates and pyrethroids [18–20]). Worryingly, there are indications that this insecticide resistance is already compromising the effectiveness of ITN and IRS control measures [17].

In this environment of ever-growing resistance, novel vector control techniques which seek to reduce mosquito populations without the need for chemical insecticides may be vital for effective malaria management [10]. Pest control via the release of sterile insects is an old idea [21] and its successes have been well-documented in other species [22, 23]—although unforeseen circumstances, such as unidentified wild breeding sites, can cause problems for these control programmes [24, 25]. The Sterile Insect Technique (SIT) may offer a means to control mosquito populations [26], and much modelling work has shown that SIT and its transgenic equivalents could be successful [27–30]. The traditional radiation-based SIT represents a form of ‘early-acting lethality’, in the sense that offspring die at the very earliest stage. However, such early-acting lethality may be undesirable: ecological studies show that larval habitats can exhibit overcompensatory density dependence, where the size of the adult population increases as larval density decreases [31]. Consequently, traditional SIT control may be ineffective [32] or, worse, even increase adult mosquito populations [33, 34]! If overcompensatory density dependence occurs, an SIT is needed which exhibits ‘late-acting lethality’, where larvae fully contribute to density-dependent competition but then die at the end of the larval stage. Advances in molecular genetics are making such late-acting SITs possible as transgenic insects carrying a dominant lethal gene have been developed [32, 35, 36], where the progeny of transgenic mosquitoes die at the end of the larval or pupal stage [37].

Information on the costs and effectiveness of different interventions are essential so that policy makers can make informed decisions. Detailed studies of the efficacy and cost-effectiveness of malaria interventions show that ACT is generally cost-effective [5, 38], and ITNs and IRSs are highly cost-effective [39–41]—though in the long term resistance is likely to render them ineffective. Therefore, even if it is technically possible to engineer transgenic insects which could reduce vector populations to a level where they can no longer sustain malaria transmission, if they are prohibitively expensive compared to the alternatives they are unlikely to change malaria management policy. For this reason, it is important to assess the cost-effectiveness of novel vector control techniques at the earliest opportunity; although such estimates require many assumptions to be made, they can still be indicative of whether policy makers should take such vector control techniques seriously or not. To date, little is known about the the cost-effectiveness of early- or late-acting SITs. Cost–benefit analyses have been performed for traditional SITs in other pest insect species [42], but not for mosquitoes. One study has examined the cost-effectiveness of using constant releases (rather than dynamically managed releases, which is investigated here) of transgenic late-acting SIT mosquitoes to reduce dengue [43]. However the cost-effectiveness of dynamic, optimized releases of transgenic late-acting SITs for malaria has not been examined, nor has any comparison been made between the cost effectiveness of early vs late-acting SIT.

Here, conventional early-acting SIT with genetics-based late-acting SIT is compared for various strengths of larval density dependence to assess how ecology affects the efficacy and cost-effectiveness of vector control. This vector control model is coupled to an epidemiological model of malaria spread between vector and host, using *P. falciparum* estimates for parameterization, under the action of ACT. The aim is to investigate both the efficacy of the combined drug and vector control strategies and identify the most cost-efficient way to deploy these strategies. To do this, two control parameters are introduced: the SIT release ratio *u* and artemisinin treatment ratio *w*, which act on the population model and disease model, respectively. Cost functions for these two parameters are developed and then weigh against the economic burden of endemic disease. This ‘optimal control framework’ is used to investigate the most cost effective way to reduce the disease burden.

## Methods

A stage-structured model of the mosquito population [44, 45] is combined with a Ross–MacDonald epidemiological model of the malaria dynamics [46–50]. The governing ordinary differential equations for the larval (*L*) and adult female (*A*) mosquito populations and the proportion of the human (*h*) and vector (*v*) populations that are infected (and infective) are

1a $$\begin{aligned} \frac{\mathrm {d}L}{\mathrm {d}t} =&\, \rho A(t) C(A,u) - f(L) L(t) - (m + \mu _L) L(t) ,\end{aligned}$$

1b $$\begin{aligned} \frac{\mathrm {d}A}{\mathrm {d}t} =&\, \frac{m}{2} L(t) - \mu _A A(t), \end{aligned}$$

1c $$\begin{aligned} \frac{\mathrm {d}h}{\mathrm {d}t} =&\, \frac{A(t)}{N} b a (1 - h(t)) v(t) - (\gamma + w(t) s) h(t), \end{aligned}$$

1d $$\begin{aligned} \frac{\mathrm {d}v}{\mathrm {d}t} =&\, b c (1 - v(t)) h(t) - \left( \mu _A + \frac{1}{A(t)}\frac{\mathrm {d}A}{\mathrm {d}t}\right) v(t), \end{aligned}$$

where $\rho$ is the mosquito oviposition rate, *m* is the rate at which larvae mature into adults (*m*/2 represents half the larvae maturing into adult females), $\mu _L$ and $\mu _A$ are the density-independent mortality rates of the larval and adult stages, respectively, *N* is the total host population, *b* is the biting rate of the vectors, *a* is the proportion of bites by infected mosquitoes on susceptible humans that produce an infected human, *c* is the proportion of bites on infected humans by a susceptible mosquitoes that produce an infected mosquito, $\gamma$ is the rate of recovery. The disease model is parameterized using *P. falciparum* estimates, see Table 1. ACT is assumed to affect a fraction *w* (the treatment proportion, or drug coverage) of infected humans through an increase in their recovery rate by an amount *s*. A continuous-time approach is chosen over a discrete-time model in order to capture the effect of control technologies and density-dependent regulation in overlapping generations. However, in order to develop the mathematics for an optimization analysis, a simplifying assumption is made such that no time-delayed effects occur, such as parasite incubation, egg development or drug-clearing times.

The function *C*(*A*, *u*) in (1) describes the effective reduction in fecundity due to mating with released sterile males (assuming the populations are well-mixed and undergo random mating), and is defined as

2 $$\begin{aligned} C(A,u) = \frac{A(t)}{A(t) + u(t)A^{*}}, \end{aligned}$$

where $A^{*}$ is the equilibrium adult female mosquito population (without vector control), and *u* is the release ratio of sterile male mosquitoes (hence $u A^{*}$ gives the instantaneous, i.e. daily, number of insects being released). The equilibrium levels of endemic *P. falciparum* infection (as a fraction of the total populations) are $h^*$ (hosts) and $v^*$ (vectors). Using Global Health Observatory data [51] for malaria prevalence in the most hard-hit African nations and estimates of sporozoite rates from Kilama et al. [52] to produce order-of-magnitude approximations for $h^*$ and $v^*$, we use the transmission efficiencies *a* and *c* to set the endemic equilibrium levels to $\sim$1%.

While there is some rudimentary information on the magnitude and type of intraspecific competition in larval *Anopheles* [31, 53], this is neither sufficiently comprehensive nor definitive. As such a relatively flexible form of density dependence [54] is chosen that has been recently used elsewhere to explore the impact of ecological intraspecific competition and genetics-based methods of vector control [34]. This form of density dependence was originally proposed by Maynard Smith and Slatkin [55] and is of the form

3 $$\begin{aligned} f(L) = \ln {\left( 1 + (\nu L)^{\beta }\right) }. \end{aligned}$$

Density dependent effects are set by a scale parameter ($\nu$) and all larval cohorts experience the same strength of density dependence (which can range from contest—resources monopolized by few individuals—to scramble—resources shared equally by all—by varying the coefficient $\beta$). Both the timing of density dependence with respect to genetic lethality mechanisms and the strength of density dependence are well-known to affect vector control programmes and can lead to ‘bad’ SIT effects [33, 34]. $\nu$ is set to satisfy the relation

4 $$\begin{aligned} \nu = \frac{m}{2 k^* N \mu _A} \left( \mathrm {e}^{\{\rho m/(2 \mu _A) - m - \mu _L\}} - 1 \right) ^{\frac{1}{\beta }}, \end{aligned}$$

to ensure that the equilibrium population $A^*$ is equivalent to $k^* N$, the number of vectors per host at equilibrium multiplied by the host population.

The population model (1a) and (1b) is modified slightly if sterile insect releases are replaced with releases of insects modified with a late-acting lethal gene [an early-acting lethal gene is adequately modelled by (1a) and (1b)]. Genetic mortality is assumed to occur at the end of the larval stage, such that all offspring will contribute fully to density-dependent mortality. Thus, the stage-structured mosquito population model takes the form

5a $$\begin{aligned} \frac{\mathrm {d}L}{\mathrm {d}t} =&\, \rho A(t) - f(L) L(t) - (m + \mu _L) L(t) \end{aligned}$$

5b $$\begin{aligned} \frac{\mathrm {d}A}{\mathrm {d}t} =&\, \frac{m}{2} L(t) C(A,u) - \mu _A A(t), \end{aligned}$$

where *C*(*A*, *u*) is as defined in (2). Model (5) will be referred to as ‘late-acting SIT’ as opposed to the ‘early-acting SIT’ (Eq. 1).

**Table 1:** Parameter definitions and values. Disease parameters use *P. falciparum* estimates Costs inflated to 2016 US$ using, where available, the 2008 baseline values from the supporting material of Alphey et al. [43]

Parameter	Description	Default value	Notes
*N*	Host population	$$2\times 10^5$$	Variable
$$k^*$$	Vectors per host at equilibrium	2	But can be as high as 200 [69, 71–73]
*b*	Mosquito bite rate	0.5 per day	[74]
*a*	Vector to human transmission efficiency	0.07	[75]
*c*	Human to vector transmission efficiency	0.1	[76, 77]
$$\gamma$$	Malaria recovery rate	1/14 per day	Variable, assuming a 2-week average with conventional treatment
*s*	Artemisinin-enhanced recovery rate	$$\frac{1}{3}\ln {10}$$ per day	$$90\%$$ success rate over 3 days [5, 78]
$$\rho$$	Per adult female oviposition rate	16 per day	[79–81]
*m*	Larval maturation rate	0.1 (0.05, 0.17)	[82]
$$\mu _A$$	Adult mosquito death rate	$$\ln \frac{10}{9}$$ per day	[73, 76, 77, 83, 84], chosen to be conservative
$$\mu _L$$	Density-independent larval death rate	0.03 per day	[85]
$$\beta$$	Strength of density dependence	0.9	Varies from $$\beta <1$$ for contest to $$\beta >1$$ for scramble
$$\nu$$	Scale of larval density-dependence	Eq. (4)	Ensures $$A^* = k^* N$$
$$\theta _1$$	Cost per infected individual	$$11.43 \gamma$$ $$(3.03 \gamma , 37.12 \gamma )$$ $ per day	Mean (min, max) from [61]
$$\theta _2$$	Cost of artemisinin therapy	$$\frac{4.61}{3}$$ $$(\frac{3.96}{3}, \frac{5.57}{3})$$ $ per day	Mean (min, max) from [5] assuming 3 day treatment
$$\theta _3 \times 10^4$$	Cost per $$10^4$$ insects released	9.11 (1.93, 25.36) $ per day	Mean (min, max) from [43, 86–89, 89, 90]
$$\psi$$	Future discounting factor	0.1/365 per day	Variable

### Optimal control

A cost functional is defined that measures the cost per capita, in US$, of disease level (*h*), artemisinin medication (*w*) and vector control (*u*) over a fixed time period $$t\in [0,T]$$:

6 $$\begin{aligned} J_{pc}[\varvec{x}(u,w)] = \int _0^T \mathrm {e}^{-\psi t} \left[ \theta _{1} h^{m_{1}} + \theta _{2} h w^{m_{2}} + \theta _{3} k^{*} u^{m_{3}}\right] \mathrm {d}t, \end{aligned}$$

where future costs are discounted at a constant rate $\psi$ to deal with opportunity costs in investing in public health control for immediate benefits [56, 57], $\varvec{x} = (L, A, h, v)^{\top }$ is the vector of state variables and the parameters $\theta _i$, $k^*$ and $\psi$ take values given in Table 1. The price factors $\theta _i$ and the cost function exponents $m_i$ govern the scale and behaviour of cost for each contributing factor, and form the (per capita) cost functions $C_h = \theta _{1} h^{m_{1}}$, $C_w = \theta _{2} h w^{m_{2}}$ and $C_u = \theta _{3} k^{*} u^{m_{3}}$ (see Appendix A: “Optimal control formulation” for more details). Drug coverage was capped at a maximum of $60\%$ ($w_{\mathrm {max}} = 0.6$); this prevents the optimization approach from recommending drug coverages close to $100\%$, as such high levels would be unachievable in reality.

The goal is to minimize the total cost of the malaria management strategy, where the controls are the insect release ratio (*u*) and the artemisinin treatment ratio (i.e. drug coverage, *w*):

7 $$\begin{aligned} \mathop {\mathop {\min }\limits _{u \ge 0} }\limits _{0 \le w \le 0.6} \{ {J_{pc}}\}. \end{aligned}$$

The method used to solve this optimisation problem is described in Appendix A: “Optimal control formulation”, along with some associated mathematical results.

## Results

### Disease elimination and extinction

**Fig. 1:**
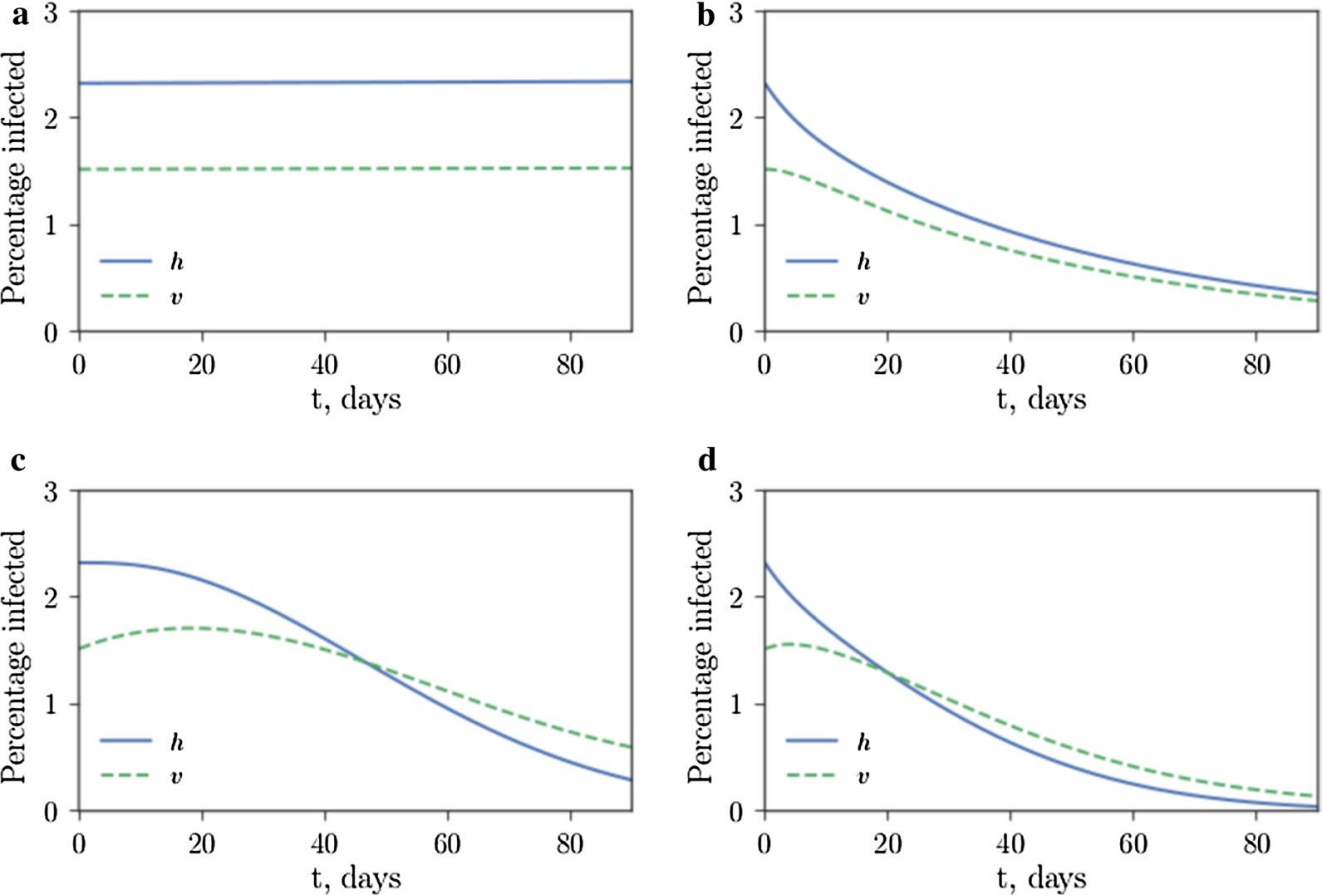
Vector control and drug therapies are most effective when used in tandem. Proportions of humans (*h*) and vectors (*v*) infected are plotted under four control scenarios. **a** no control, $w=0$, $u=0$; **b** only artemisinin treatment, $w=0.05$ ($5\%$ drug coverage), $u=0$; **c** only vector control, $w=0$, $u=0.2$ (releasing modified males at a rate of $20\%$ of the wild male populations per day); **d** both artemisinin treatment and vector control, $w=0.05$, $u=0.2$. The total number of infected mosquitoes at time $t=90$ days for each scenario is **a** 6125 **b** 1145 **c** 238 **d** 55; the vector control suppresses the vector population significantly. Early-acting SIT is assumed

Model simulations (based on the parameters in Table 1) show, as expected, that in the absence of control, the proportion of infected individuals, *h*, and infected vectors, *v*, will reach an endemic level (Fig. 1a). Introducing drug control, whereby individuals recover at an enhanced rate, can reduce endemic levels of disease in hosts (Fig. 1b). Mosquito vector control (through population suppression technologies such as SIT or genetic engineering) can effectively reduce disease (Fig. 1c); by combining both vector control and drug-based therapies, disease spread is reduced and disease control is most effective (Fig. 1d).

**Fig. 2:**
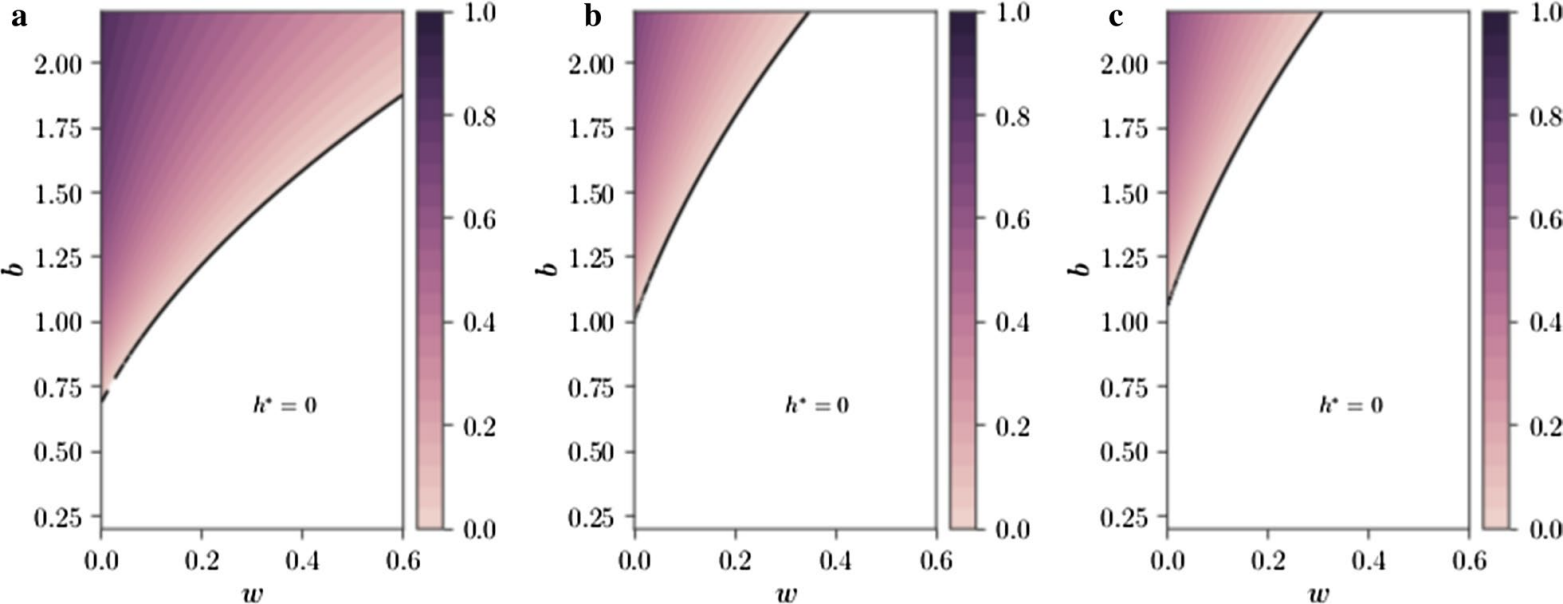
Vector control broadens disease-free parameter space, causing disease fade-out even for high biting rates. The level of endemic disease, $h^*$, across *b*–*w* parameter space (biting rate–drug treatment proportion) with **a** no vector control, **b** early-acting SIT releases and **c** late-acting SIT releases. The release ratio in **b** and **c** is $u=0.1$ (daily releases of $10\%$ of the wild male population), with the ratio being of the current vector population *A*(*t*) rather than the equilibrium population $A^*$ (elsewhere in the paper the ratio of $A^*$ is used). Disease-free regions of parameter space are coloured white. Other parameters as in Table 1

**Fig. 3:**
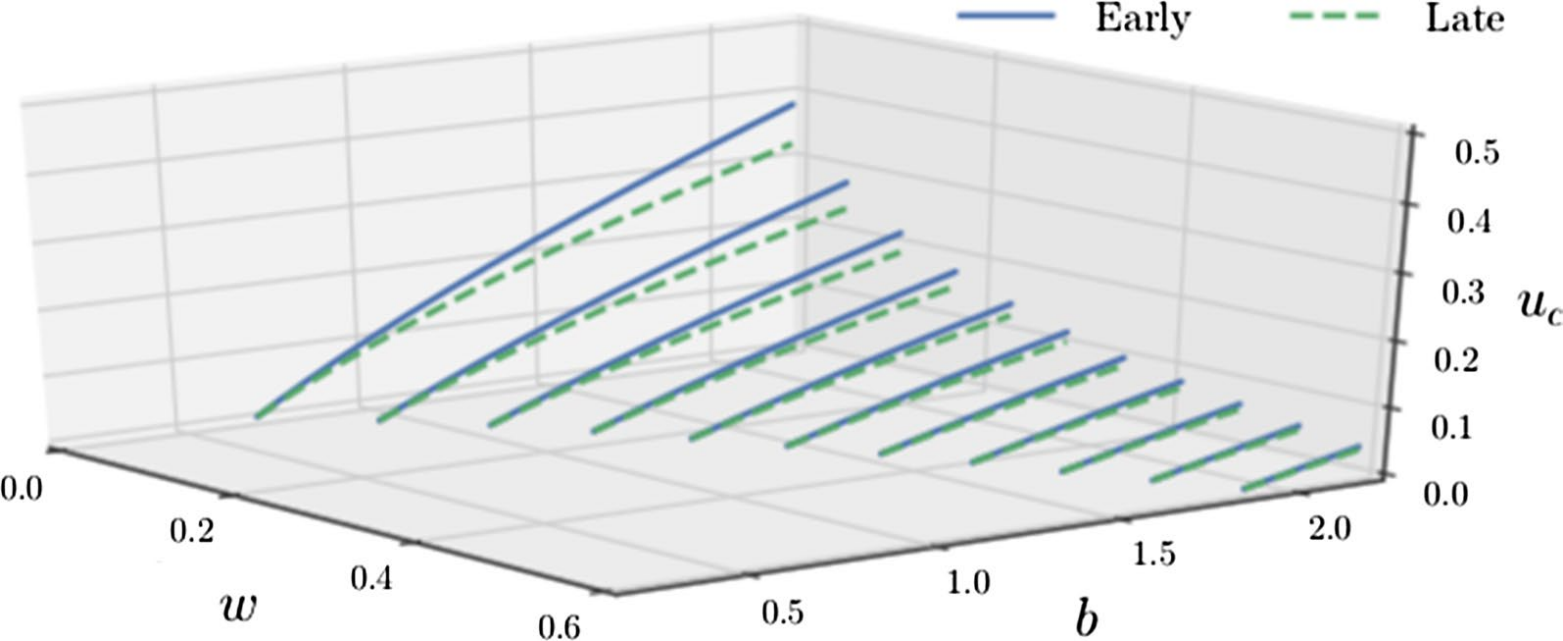
Vector control releases can achieve disease fade-out for sparse drug coverage when biting rates are below $b<1.2$. The critical release ratio $u=u_{\mathrm {c}}$ that leads to disease fadeout ($h^*\rightarrow 0$) for a given treatment proportion *w* as the mosquito biting rate *b* varies, as calculated using (46) and (47). The release ratio *u* is of the current vector population *A*(*t*) rather than the equilibrium population $A^*$ (elsewhere in the paper the ratio of $A^*$ is used). Early and late SIT are compared; all other parameters as in Table 1

It is usual to discuss values of the basic reproductive ratio ($R_0$) of disease when analysing epidemiological models. The value of $R_0$ is a static measure of the speed of disease spread in a naïve environment [49, 58]. Effective reproductive ratios can be constructed which take account of growing host populations [59] or heterogenous environments [60], but these still cannot capture the dynamic effect of vector control via SIT or transgenic insect releases. Rather than investigating how quickly a disease might invade a disease-free population (a situation in which SIT releases would not be in use), here it is investigated how far below the control-free endemic equilibrium the prevalence of disease is pushed by vector control (a situation in which insect releases would be called for). The equilibria of the system with and without control are given in Appendix B: “[Sec Sec11]”. If the application of control (drug-based and vector) takes the new equilibrium of *h* to zero, then that point in parameter space is considered disease-free. In the classic control-free, disease-naïve scenario, the $R_0$ value depends upon the square of biting rate *b*, and is linear in *a* and *c* (see Appendix B: “[Sec Sec11]”). Thus, *b* is of greater importance to disease spread than the other parameters that constitute $R_0$ (*a*, *c*, *k*, $\gamma$ and $\mu _A$). Changes in *b* will therefore exert relatively large changes in disease prevalence, which can be balanced by implementation of drug therapies (*w*) and vector control. Therefore, disease-free regions that occur in *b*–*w* parameter space are investigated.

For the constant release ratio $u=0.25$ (where here releases are made in a ratio to the current vector population *A*(*t*) rather than the equilibrium population $A^*$, a comparison first made in [37]) the disease-free region of *b*–*w* space is increased considerably (Fig. 2), and the severity of the endemic disease away from the disease-free region is reduced for both early- and late-acting SIT (compare Fig. 2a with Fig. 2b, c). The greatest effect of vector control on $h^*$ is seen at $w=1$ (Fig. 2b, c), showing that the most effective way to combat endemic disease is through a combination of vector control and drug-based therapies. Late-acting SIT is seen to out-perform early-acting SIT at the same release ratio.

The critical release ratio $u = u_{\mathrm {c}}$ (when releasing *uA*(*t*) insects rather than $u A^*$ insects) that leads to disease fadeout ($h^*\rightarrow 0$) may be found (see Appendix B.2: “Equilibria and the effect of control”). For biting rates below $b\approx 1$ (i.e. vectors blood feed as frequently as once per day), manageable vector control releases, $u<0.1$, can be used to cause disease fadeout when drug coverage is sparse, $w<0.2$ (Fig. 3). For high biting rates, $b>1.2$, the use of drug therapies can substantially reduce the critical release ratio, for example for $b=2$ maintaining drug coverage of $60\%$ can reduce the required mosquito release rates from $30\%$ of the wild male population daily to under $5\%$.

### Optimal control

**Fig. 4:**
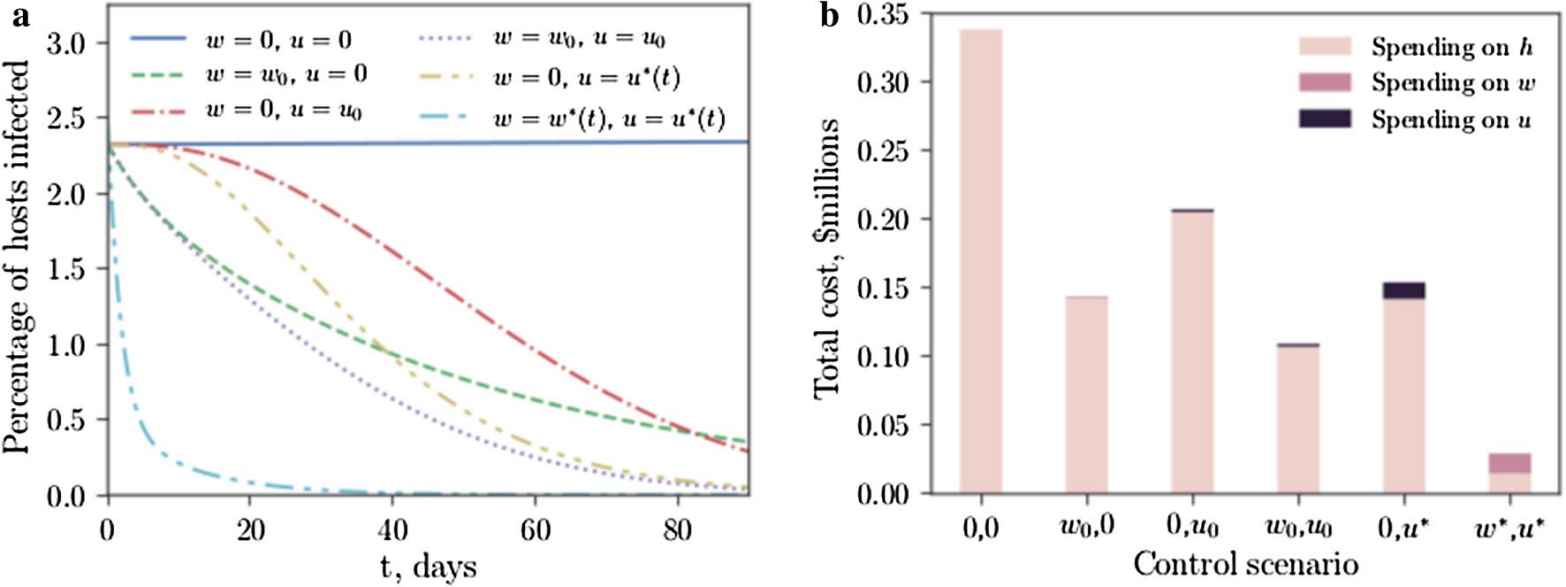
Optimal strategies for releases and drug treatments can substantially reduce the cost of managing malaria. **a** The proportion of infected humans under six control scenarios (where *w* is the host treatment proportion and *u* is the insect release ratio with respect to the wild vector population): no control (*x*-axis label “0,0”), $w=w_0$, $u=0$ (*x*-axis label “$w_0$,0”), $w=0$, $u=u_0$ (*x*-axis label “0,$u_0$”), $w=w_0$, $u=u_0$ (*x*-axis label “$w_0$,$u_0$”), $w=0$, $u=u^*(t)$ (*x*-axis label “0,$u^*$”), $w=w^*(t)$, $u=u^*(t)$ (*x*-axis label “$w^*$,$u^*$”), where $w_0 = 0.05$, $u_0 = 0.2$ ($5\%$ drug coverage and releasing $20\%$ of the wild male population per day) and $w^*$, $u^*$ are the optimal control strategies defined in (18) and (29), respectively. **b** The total cost of the scenario, including spending on traditional healthcare (*h*), spending on artemisinin treatment (*w*) and spending on insect releases (*u*). Early-acting SIT is assumed. Quadratic cost functions of *h*, *w* and *u* are assumed

Control regimes may more efficiently (with respect to time and cost) combat disease when the optimal control solutions (described in Appendix A.1: “Optimality conditions”) are implemented (Fig. 4). In Fig. 4a the four naïve control scenarios of Fig. 1 are compared with an optimal vector control strategy $u^*(t)$, (29), with no artemisinin treatment, and an optimal control strategy using both vector control and drug therapies $w^*(t)$, (18). The breakdown of costs (using the pricing parameters given in Table 1) neglects the one-off capital investment used to a construct insect-rearing facility (the facility could be used again for future control efforts—the operational costs for a single season are presented here).

**Fig. 5:**
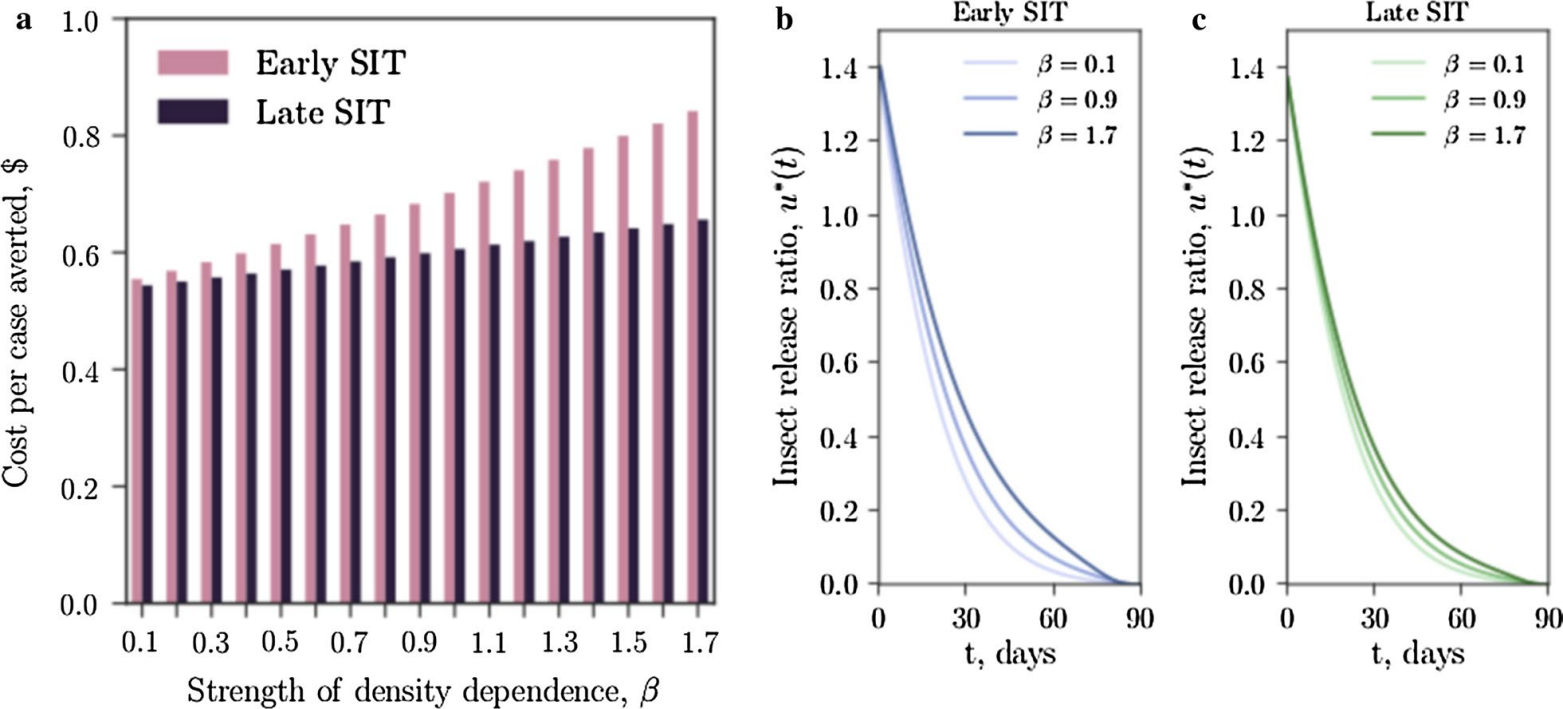
Late-acting lethality suppresses the effects of over-compensatory larval density dependence. **a** Cost per case averted (excluding initial capital investment in construction) for optimized release strategies of early-acting and late-acting SIT, for the parameters given in Table 1, as the strength of the larval density dependence $\beta$ is increased. The density dependence runs from contest for $\beta < 1$ to scramble for $\beta > 1$. The number of cases averted is approximated by ([mean no. with no control − mean no. with control]/average duration of disease) $\times$ no. of days). The change in the optimal release strategy is shown for **b** early SIT, and **c** late SIT for three specific values of $\beta$. Artemisinin treatment is not used

When density-dependent effects at the larval stage are weak, early- and late-acting SIT technologies are similar in efficacy; when density-dependent effects are strong, the two technologies diverge and late-acting SIT is shown to be the most cost-effective (Fig. 5). Although the form of the optimal control strategies for early- and late-acting SIT are similar (Fig. 5b, c), the enhanced efficacy of late-acting SIT due to density-dependent mortality means lower release ratios produce optimal results, requiring facilities with lower weekly rearing capacities.

**Fig. 6:**
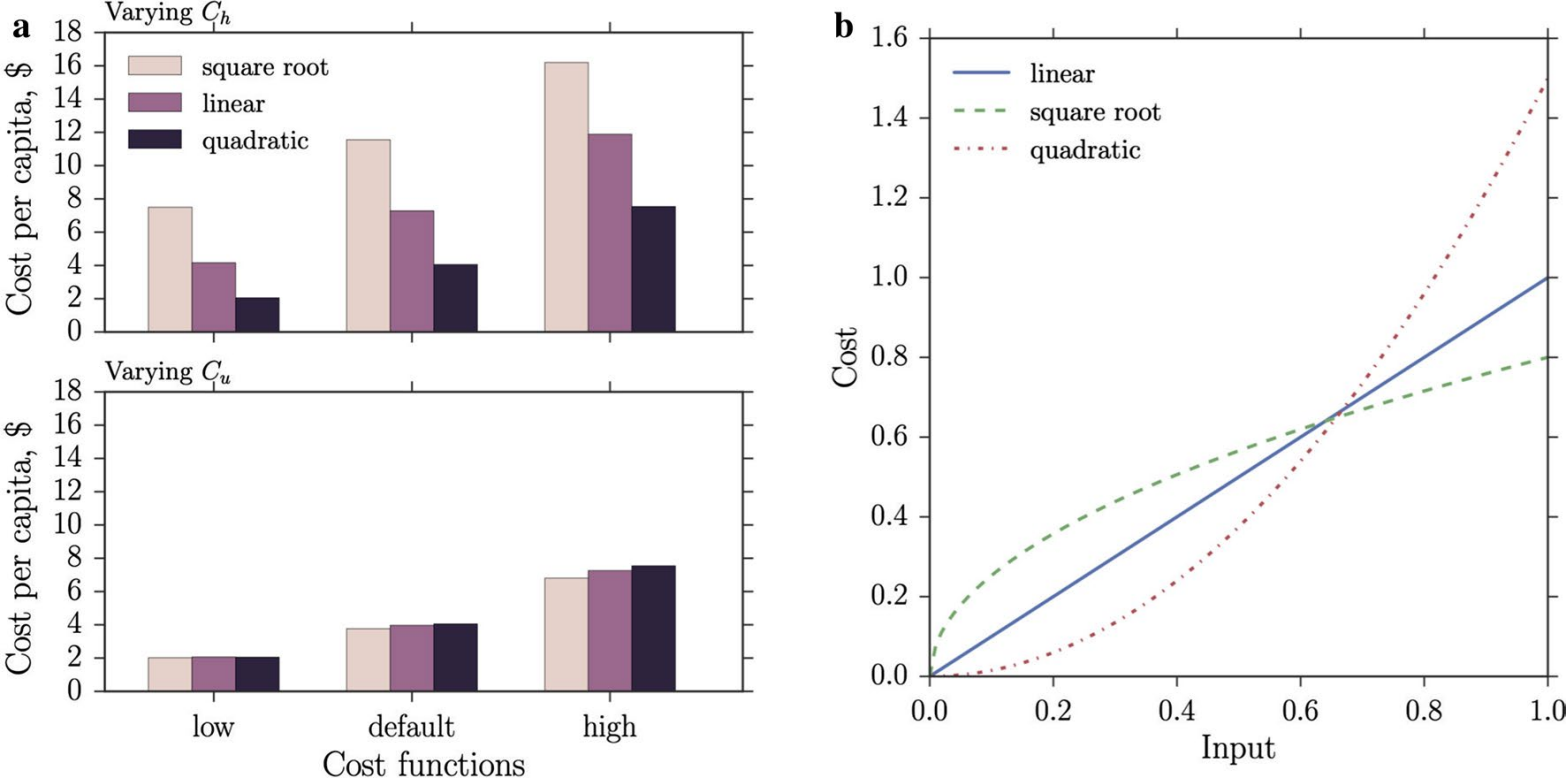
Vector control costs, unlike medical care costs, are insensitive to the form of cost function. **a** Cost per capita for the optimal strategy over a 40 day control period for three cost functions: linear, quadratic and square root; and three epidemiological parameter sets: low, medium and high (for low $a,b,c,k^* = 75\%$ of medium, for high $a,b,c,k^* = 125\%$ of medium). In the top figure of **a** the cost function $C_h$ is varied while keeping $C_u$ and $C_w$ quadratic ($m_2 = m_3 = 2$); in the bottom figure the cost function $C_u$ is varied while keeping $C_h$ and $C_w$ quadratic ($m_1 = m_2 = 2$). The number of cases averted is approximated by ([mean no. with no control - mean no. with control]/average duration of disease) $\times$ no. of days). **b** A sketch of the three cost functions $\hat{\theta }_i (\cdot )$ (linear), $\hat{\theta }_i (\cdot )^{1/2}$ (square root) and $\hat{\theta }_i (\cdot )^2$ (quadratic), where the altered price $\hat{\theta }_i$ that multiplies the cost function is: 0.8 (square root), 1 (linear), 1.5 (quadratic). Late-acting SIT is used

**Fig. 7:**
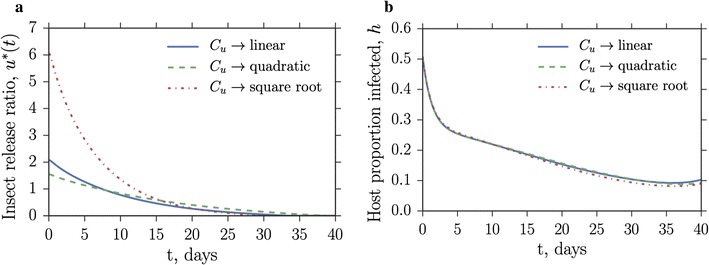
Increasing the initial insect release ratio has diminishing returns on the effect on disease prevalence. Example solutions from the default epidemiological parameter set of Fig. 6a when $C_u$ is varied through square root, linear and quadratic.** a** Shows the optimal vector release strategy, **b** shows the resulting suppression of disease in the host population. Late-acting SIT is used. Other parameters as in Table 1

The general cost function exponents in (6) for *h* and *u* allow for possible nonlinearities in cost to be investigated separately for each variable; the exponent of $C_w$ is restricted to $m_2>1$ as outlined in the discussion in Appendix A.1: “Optimality conditions”. Figure 6a shows that reducing the cost exponent $m_1$ of $C_h$ increases the cost per capita over the control period for the combined vector control and drug therapies optimal strategy. An exponent $m_1<1$ describes a situation where treating a small proportion of the population is expensive, but the cost levels off at higher treatment proportions as economies of scale take effect; conversely, an exponent $m_1>1$ describes a situation where treating a reasonable proportion of the population can be done cheaply, but the price steepens for high proportions, capturing the fact that clinics may exceed capacity, or some sections of the population may be more difficult to reach (see Fig. 6b). Changing the cost function of the vector control variable *u* has a relatively small effect due to the low cost associated with releasing a large number of mosquitoes (Fig. 6a, lower plot). The optimal strategy for vector control is generally to swamp the wild population at the beginning of the control period, and then reduce the releases as the control takes effect (Fig. 7a). For a square root cost function it is very cheap to release a large number of mosquitoes (which is mathematically optimal), but the greater the initial influx of released mosquitoes, the greater the capacity must be of the rearing facility. This can lead to construction costs that dwarf the control-free costs of conventional treatment of endemic malaria over a single season. In fact, diminishing returns are seen in the effect on *h* of increasing the initial release ratio *u* (Fig. 7b), as the square root cost function solution for *u* causes only a slight decrease in *h* over the control period when compared to linear or quadratic cost functions, for which the optimal release strategies are initially more conservative.

### Concentrated or distributed control?

**Fig. 8:**
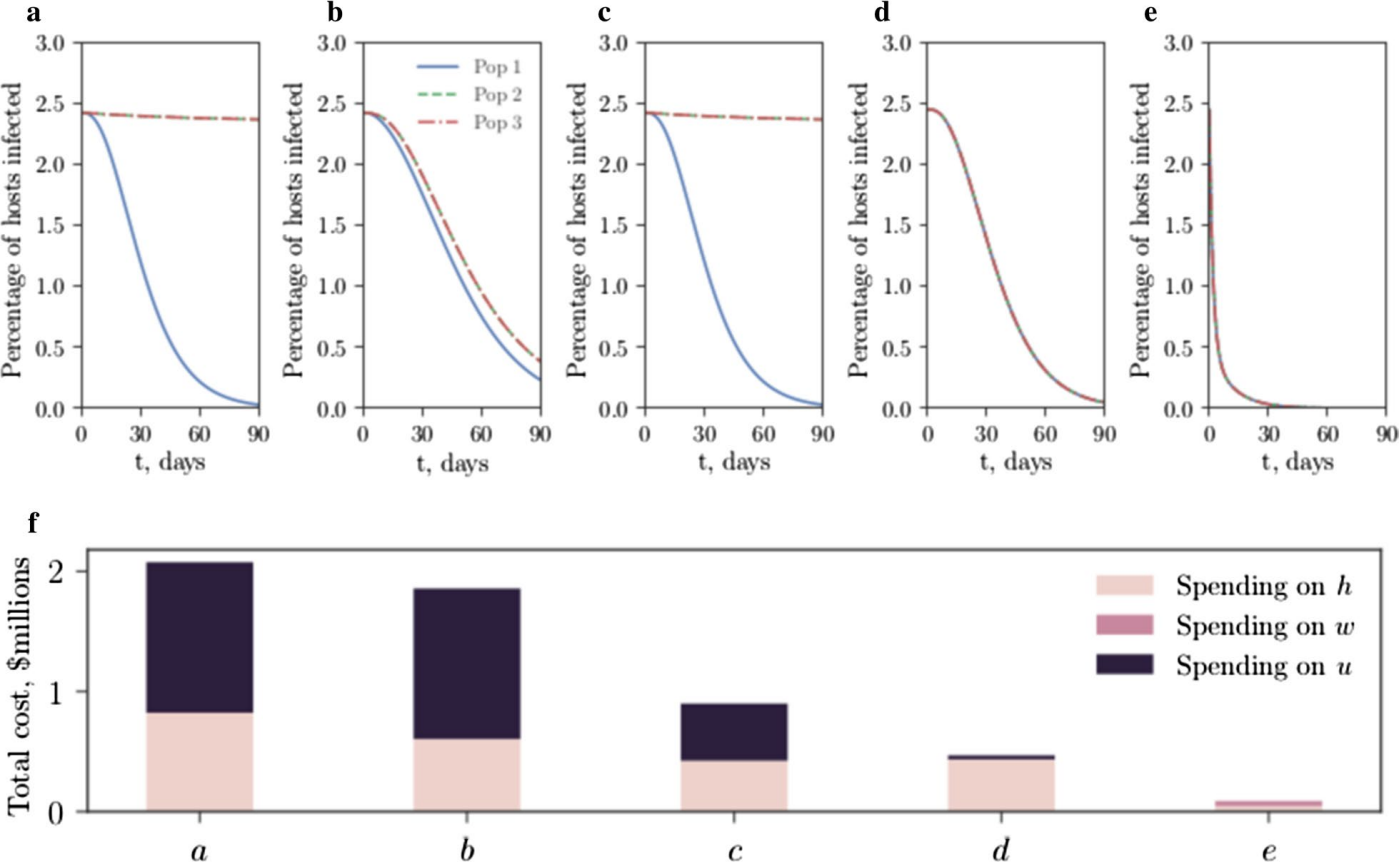
Migration can harm concentrated control efforts; distributed vector control with combined drug treatment is optimal. The proportion of hosts infected in each of three identical populations with SIT release ratios $u_1$, $u_2$ and $u_3$, where migration is $10\%$ between nodes except in **a** where there is no migration. **a** $u_1=3$, $u_2 = u_3 = w_i = 0$; **b** $u_1=3$, $u_2 = u_3 = w_i = 0$ (concentrated control); **c** $u_1 = u_2 = u_3 = 1$. $w_i = 0$ (distributed control); **d** $u_i(t) = u_i^*(t)$, $w_i = 0$ (optimal vector control); **e** $u_i(t) = u^*(t)$, $w_i(t) = w_i^*(t)$ (optimal combined drug treatment and vector control). **f** Cost breakdowns for the five scenarios plotted above. All parameters as in Table 1. Late-acting lethality is used

A small network of three equally spaced populations is established to investigate the effects of migration on the efficacy of vector control, Fig. 8 (see Appendix C: “A network model” for model details). Three methods of vector control are compared: a concentrated control approach, where one population is subjected to a constant high SIT release ratio $u = 3$, a distributed control approach, where each population is subjected to a constant low SIT release ratio $u=1$ (the total number of released insects is the same in these two cases), and an optimal vector control approach for each population, with and without combined optimized drug treatment. As SIT methods are an area-wide control strategy, mosquito dispersal, although often limited by energy resources, from an uncontrolled population can damage the efficacy of vector control in a focal population (Fig. 8a, b). Distributing control efforts across populations is shown to be a more economically efficient and effective vector intervention strategy than focusing on a single disease hot-spot (Fig. 8b, c). Optimal vector control strategies (rather than constant release ratios) can reduce total expenditure, but the greatest monetary saving is made when combining optimal vector control with optimal drug treatment (Fig. 8d–f).

## Discussion and conclusions

Here a novel framework that links disease dynamics, an ecological mosquito population model and an economic optimal control formulation has been developed. An optimal control framework was used to assess the efficacy and cost-effectiveness of combining ACT with two novel vector control technologies: releasing either sterile mosquitoes or transgenic mosquitoes carrying a late-acting lethal gene. In all cases, the transgenic late-acting mosquitoes are more cost-effective than the early-acting sterile insects. This is because the transgenic mosquito larvae compete with the wild mosquito larvae; the resulting competition increases larval mortality which reduces the wild type adult mosquito population. Immigration of vectors from uncontrolled populations can damage a concentrated control effort, and found that it is optimal to use distributed control strategies combined with drug treatments. Results from this analysis revealed that the combined drug and vector control interventions have a cost per case averted of $$\sim \$1$$ (2016 US$) if construction costs are neglected, or $$\sim \$4$$ if construction costs are included in the control costs for a single season. These estimates are necessarily based on assumptions about the cost of transgenic releases and construction costs of insect rearing facilities, which are uncertain given that this technique is at such an early stage of development. Nevertheless, even if the real costs are double or even triple those predicted here, they compare extremely favourably with the economic costs of the current leading malaria control methods [61].

Boundaries of disease-free regions of (biting rate–drug treatment ratio) parameter space were found by calculating post-control population equilibria. This method was chosen over the usual $R_0$ calculations for two reasons: (i) the dynamic effect of vector control could not be captured by the static $R_0$ value, (ii) the disease-naïve invasion basis of $R_0$ does not fit well with the goal of assessing the control of endemic disease. Vector control has a positive effect on the size of disease-free regions and can reduce the prevalence of disease outside of these regions (Fig. 2). The use of drug therapies can significantly lower the critical release ratio required to eradicate disease even for high vector biting rates (Fig. 3), showing that combining control methods can be an effective disease management strategy.

Drug therapies act to reduce the level of endemicity and suppress the initial spread of disease, while vector control combats disease over time and continuously reduces infection levels towards zero (Fig. 4a). Optimal control strategies follow a robust pattern, indicating that a high initial release ratio is necessary for cost-effective vector control (Fig. 7a). A large proportion of the cost of a single-season control regime is taken up by the construction of the insect rearing facility, but these costs were neglected as it was assumed that vector control efforts will be repeated every season using the same infrastructure. Multi-season control efforts will benefit more from optimal vector release strategies as returns are made from the construction investment in the form of efficiency savings and reductions in the burden of conventional health care. There is an opportunity to extend this work by including construction costs in the optimization procedure which, while not straightforward, would enable an investigation of the (possibly competing) dual desires to keep initial costs down but strive for long-term cost efficiency.

An investigation of nonlinear (concave or convex) cost structures was carried out to model different real-world cost responses (Fig. 6). For concave-cost SIT releases, diminishing returns were found in the effect on the infection level when swamping the wild population with large numbers of lab-reared insects (Fig. 7). This suggests that it is wise to be conservative with maximum facility output requirements and highlights the importance of checking (prior to implementation) the cost-effectiveness of optimal strategies derived using a variety of cost structures. The exact form of the cost functions could be better-informed with additional, or more specific, data. Alternatively, to develop better comparisons between different regions, costs associated with endemic malaria and costs of controls might be weighted using GDP per capita, or through a purchasing power parity scheme (such as the Geary–Khamis dollar; [62]). Vector control costing could be made more reliable if information on *Anopheles gambiae* facility operational costs (insect rearing, staff pay, heating, materials etc.) was available, rather than inferring from data about facilities rearing different species. Information on the cost of surveying (needed for estimating current vector populations and for effectively targeting releases) would also be beneficial.

A network population model with migration between nodes weighted by relative distance allowed optimal control in a spatial environment with identical host populations to be studied. Migration from uncontrolled nodes was seen to lessen the effectiveness of control on neighbouring nodes (Fig. 8a, b). Distributed control at all pertinent nodes was shown to be the most efficient use of resources (Fig. 8c). While it is difficult to parameterize a model of mosquito movement (though values between 4–$24\%$ migration between neighbouring villages are reported in [63]), the spatial ecology of *A. gambiae* will undoubtedly be crucial in determining the spread or containment of control efforts. The network model proposed here could be improved by including human movement [64] as a means to spread infection. Using the optimal control framework in such a model would require reformulating cost functions capable of capturing heterogeneous populations, which is beyond the scope of this study.

The ecological and epidemiological models used here are simplified to allow an optimal control framework to be established. Worthwhile extensions to this work would be the addition of a disease incubation period in either the vector or host; use of time delays between egg laying and hatching and pupation and emergence; modelling possible periods of immunity to reinfection conferred by artemisinin treatments (if the prophylactic period is greater than the infectious period; [65]), although the relationship between drug half-life and resistance development is complex [66, 67]; a relaxation of the assumptions of random mating between wild and released mosquitoes (unsuccessful mating has been a serious impediment to codling moth control programmes; [68]) and of perfect sterility and lethality; the inclusion of fitness costs for sterile or transgenic mosquitoes. Finally, that other vectors of malaria (e.g. *Anopheles funestus*) can be important [69], and interspecific competition may lead to a shift of primary vector if *A. gambiae* is suppressed [70]. A model incorporating the ecologies of two vector species would shed light on the cost-effectiveness of control if primary vector replacement occurs.

## Appendix A: Optimal control formulation

Optimal control strategies for disease control rely on defining objective functions from which the cost-effectiveness of interventions can be evaluated. Here the costs of treating infected individuals, the costs of drug based therapies and the costs of SIT mosquito releases are explored, and an objective functional is defined to explore different cost structures (costs are linear, costs are convex, costs are concave).

Two control parameters are present in the model (1): the release ratio *u* and the proportion medicated *w* as optimal control strategy to manage the spread of malaria is required. This strategy would minimize the socioeconomic burden of malaria, the cost of artemisinin medication and the cost of vector control via sterile insect release. If the cost of direct and indirect health care and lost productivity due to a single case of malaria is $\theta _{1}$ per day, then the daily cost of having a proportion *h* of a population of size *N* infected is $\theta _{1} N h,$ which has units of dollars per day. To capture the potential nonlinearity of cost, the cost function is defined as $C_{h} = \theta _{1} N h^{m_{1}},$ which also has units of dollars per day. The exponent $0<m_{1}\le 2$ affects only the dimensionless quantity *h*, and varying $m_1$ over its range allows us to investigate cost structures that are linear, convex or concave. Similarly, if the dollar cost of medicating a single patient with artemisinin-based therapy is $\theta _{2}$ per day, then the daily cost of medicating a proportion *w* of an infected population of size *Nh* is $\theta _{2} N h w.$ As before, a cost function is defined, $C_{w} = \theta _{2} N h w^{m_{2}},$ which has units of dollars per day and where the exponent $0<m_{2}\le 2$ affects only the dimensionless quantity *w*. Finally, if the cost of rearing and releasing one (or 100, or 1000, depending on population units) sterile or GM insect per day is $\theta _{3},$ then releasing a proportion *u* of the equilibrium vector population $A^{*}$ daily will cost $\theta _{3} A^{*} u$ per day. Again, to allow for possible nonlinearity in costing, the cost function is defined as $C_{u} = \theta _{3} A^{*} u^{m_{3}},$ which has units of dollars per day independent of the value of the exponent $0<m_{3}\le 2,$ which affects only the dimensionless quantity *u*.

Using the cost functions defined above, a cost functional is defined

8 $$\begin{aligned} J[\varvec{x}(u,w)] = \int _0^T \mathrm {e}^{-\psi t} \left[ \theta _{1} N h^{m_{1}} + \theta _{2} N h w^{m_{2}} + \theta _{3} A^{*} u^{m_{3}}\right] \mathrm {d}t, \end{aligned}$$

where future costs are discounted at a constant rate $\psi$ to deal with opportunity costs in investing in public health control for immediate benefits [56], and $\varvec{x} = (L, A, h, v)^{\top }$ is the vector of state variables. The cost function *J* measures the total cost of disease, artemisinin medication and vector control over a fixed time period *T*, and has units of dollars. From (8) a new functional is defined $J_{pc} = J/N$, which measures the total cost per capita:

9 $$\begin{aligned} J_{pc}[\varvec{x}(u,w)] = \int _0^T \mathrm {e}^{-\psi t} \left[ \theta _{1} h^{m_{1}} + \theta _{2} h w^{m_{2}} + \theta _{3} k^{*} u^{m_{3}}\right] \mathrm {d}t, \end{aligned}$$

where $k^{*} = A^{*}/N$ is the number of vectors per host at control-free equilibrium. The goal, then, is to minimize the total cost of the malaria management strategy:

10 $$\begin{aligned} \mathop {\mathop {\min }\limits _{u \ge 0} }\limits _{0 \le w \le 0.6} \{ {J_{pc}}\} . \end{aligned}$$

To solve the optimization problem (1) (9) and (10), the Hamiltonian and adjoint system (see below) are formed and analytical expressions for the characterizations of the optimal controls $u^*(t)$ and $w^*(t)$ are derived that minimize the Hamiltonian (see Appendix A.1: “Optimality conditions”). To solve the system, the forward–backward sweep method is used with Runge–Kutta integration schemes [91].

For early-acting SIT, the Hamiltonian is

11 $$\begin{aligned} H =&\,\, \mathrm {e}^{-\psi t} \left[ \theta _{1} h^{m_{1}} + \theta _{2} h w^{m_{2}} + \theta _{3} k^{*} u^{m_{3}}\right] + \lambda _{1}\left[ \rho A C(A,u)\nonumber \right. \\&\left. - f(L) L - (m + \mu _L) L\right] + \lambda _{2}\left[ \frac{m}{2} L - \mu _A A\right] \nonumber \\&\,+ \lambda _{3}\left[ \frac{A}{N} b a (1 - h) v - (\gamma + w s) h\right] + \lambda _{4}\left[ b c (1 - v) h - \frac{m L}{2 A} v\right] , \end{aligned}$$

where $\lambda _{i}(t)$ are the adjoint variables. Each adjoint variable $\lambda _{i}$ satisfies an equation found by differentiating the Hamiltonian with respect to its corresponding state variable $x_{i}$ and negating:

12a $$\begin{aligned} \frac{\mathrm {d}\lambda _1}{\mathrm {d}t} =&\, -\frac{\partial H}{\partial L} = \lambda _{1}(f'(L)L + f(L) + m + \mu _{L}) - \frac{1}{2}\lambda _{2}m + \lambda _{4}\frac{m v}{2 A}, \end{aligned}$$

12b $$\begin{aligned} \frac{\mathrm {d}\lambda _{2}}{\mathrm {d}t} =&\, -\frac{\partial H}{\partial A} = -\lambda _{1}\rho C(A,u) \left( 1 + A \frac{\partial }{\partial A}\log {C(A,u)}\right) \nonumber \\&+ \lambda _{2}\mu _{A} - \lambda _{3}\frac{b a}{N}(1 - h) v - \lambda _{4}\frac{m L v}{2 A^{2}}, \end{aligned}$$

12c $$\begin{aligned} \frac{\mathrm {d}\lambda _{3}}{\mathrm {d}t} =&\, -\frac{\partial H}{\partial h} = -\mathrm {e}^{-\psi t}(m_{1} \theta _{1} h^{m_{1}-1} + \theta _{2} w^{m_{2}}) + \lambda _{3}\left( \frac{A}{N} b a v + \gamma + w s\right) - \lambda _{4}b c (1 - v), \end{aligned}$$

12d $$\begin{aligned} \frac{\mathrm {d}\lambda _{4}}{\mathrm {d}t} =&\, -\frac{\partial H}{\partial v} = -\lambda _{3}\frac{A}{N} b a (1 - h) + \lambda _{4}\left( b c h + \frac{m L}{2 A}\right) . \end{aligned}$$

The system (12) is closed by the transversality condition

13 $$\begin{aligned} \lambda _{i}(T) = 0. \end{aligned}$$

For the late-acting SIT, which prevents larvae from reaching the adult stage [the dynamics of which are captured in (5)], the Hamiltonian is

14 $$\begin{aligned} H =&\,\, \mathrm {e}^{-\psi t} \left[ \theta _{1} h^{m_{1}} + \theta _{2} h w^{m_{2}} + \theta _{3} k^{*} u^{m_{3}}\right] + \lambda _{1}\left[ \rho A - f(L) L - (m + \mu _L) L\right] \nonumber \\&+\lambda _{2}\left[ \frac{m}{2} L C(A,u) - \mu _A A\right] \nonumber \\&\,+ \lambda _{3}\left[ \frac{A}{N} b a (1 - h) v - (\gamma + w s) h\right] + \lambda _{4}\left[ b c (1 - v) h - \frac{m L}{2 A} C(A,u) v\right] , \end{aligned}$$

and the adjoint system is

15a $$\begin{aligned} \frac{\mathrm {d}\lambda _1}{\mathrm {d}t} =&\, -\frac{\partial H}{\partial L} = \lambda _{1}(f'(L)L + f(L) + m + \mu _{L}) - \frac{1}{2} \lambda _{2} m C(A,u) + \lambda _{4}\frac{m}{2 A} C(A,u) v, \end{aligned}$$

15b $$\begin{aligned} \frac{\mathrm {d}\lambda _{2}}{\mathrm {d}t} &=-\frac{\partial H}{\partial A} = -\lambda _{1}\rho - \lambda _{2}\left( \frac{m}{2} L \frac{\partial }{\partial A}C(A,u) - \mu _{A}\right) - \lambda _{3}\frac{b a}{N}(1 - h) v \nonumber \\& \quad - \lambda _{4}\frac{m L v}{2 A^{2}}C(A,u)\left( 1 - A \frac{\partial }{\partial A}\log {C(A,u)}\right) , \end{aligned}$$

15c $$\begin{aligned} \frac{\mathrm {d}\lambda _{3}}{\mathrm {d}t} =&\, -\frac{\partial H}{\partial h} = -\mathrm {e}^{-\psi t}(m_{1} \theta _{1} h^{m_{1}-1} + \theta _{2} w^{m_{2}}) + \lambda _{3}\left( \frac{A}{N} b a v + \gamma + w s\right) - \lambda _{4}b c (1 - v), \end{aligned}$$

15d $$\begin{aligned} \frac{\mathrm {d}\lambda _{4}}{\mathrm {d}t} =&\, -\frac{\partial H}{\partial v} = -\lambda _{3}\frac{A}{N} b a (1 - h) + \lambda _{4}\left( b c h + \frac{m L}{2 A}C(A,u)\right) . \end{aligned}$$

with boundary conditions as defined in (13).

### A.1 Optimality conditions

The optimal controls $(u^{*}, w^{*})$ minimize the Hamiltonian *H*. Thus,

16 $$\begin{aligned} \frac{\partial H}{\partial u} = 0 \quad \mathrm {at} \,\, u = u^{*},\quad\frac{\partial H}{\partial w} = 0 \quad \mathrm {at} \,\, w = w^{*}. \end{aligned}$$

For the early-acting SIT, artemisinin control is investigated. When $m_{2}\ne 1,$

17 $$\begin{aligned} \frac{\partial H}{\partial w} = m_{2} \theta _{2} h w^{m_{2}-1}\mathrm {e}^{-\psi t} - \lambda _{3} s h = 0 \qquad \mathrm {at} \,\, w = w^{*}. \end{aligned}$$

The optimal control $w^{*}$ may then be found:

18 $$\begin{aligned} w^{*} = \left( \frac{\lambda _{3} s}{m_{2} \theta _{2}}\mathrm {e}^{\psi t}\right) ^{\frac{1}{m_{2}-1}}. \end{aligned}$$

The second derivative of *H* indicates whether the solution is a maximum or a minimum:

19 $$\begin{aligned} \frac{\partial ^2 H}{\partial w ^2} = (m_2 - 1) m_2 \theta _2 h w^{m_2 - 2} \mathrm {e}^{\psi t}; \end{aligned}$$

this is only positive if $m_2>1,$ so the solution (18) is restricted to cost functions with $m_2>1.$

For the vector control, consider the case $m_{3}=1.$ Differentiating the Hamiltonian for early-acting SIT produces

20 $$\begin{aligned} \frac{\partial H}{\partial u} = m_{3} \theta _{3}\frac{A^{*}}{N}\mathrm {e}^{-\psi t} - \frac{\lambda _{1} \rho A^{2} A^{*}}{(A + A^{*}u)^{2}} = 0 \qquad \mathrm {at} \,\, u = u^{*}, \end{aligned}$$

which may be rearranged to form a quadratic in *u* and subsequently solved to give

21 $$\begin{aligned} u^{*} = - A_{r} \pm \sqrt{\chi }, \end{aligned}$$

where $$A_{r} = A/A^{*}$$ and

22 $$\begin{aligned} \chi = A_{r}^{2} \frac{\lambda _{1} \rho N}{m_{3} \theta _{3}}\mathrm {e}^{\psi t}. \end{aligned}$$

The concavity condition holds for (21), as $\frac{\partial ^2 H}{\partial u ^2}>0.$ For late-acting SIT, the form (21) still holds but with

23 $$\begin{aligned} \chi = \frac{m L A_{r}^{2}A^{*}}{2 m_{3} \theta _{3} k^{*}}\mathrm {e}^{\psi t} ( \lambda _2 A - \lambda _4 v ). \end{aligned}$$

For the case $m_{3}\ne 1,$ differentiating the Hamiltonian leads to a rather more complicated equation for the characterization of $u^{*.}$ For the quadratic cost function $m_3 = 2,$ the derivative of the Hamiltonian for early-acting SIT is

24 $$\begin{aligned} \frac{\partial H}{\partial u} = 2\theta _{3} u \frac{A^{*}}{N}\mathrm {e}^{-\psi t} - \frac{\lambda _{1} \rho A^{2} A^{*}}{(A + A^{*}u)^{2}} = 0 \qquad \mathrm {at} \,\, u = u^{*}. \end{aligned}$$

Equation (24) may be rearranged to form a cubic in *u*:

25 $$\begin{aligned} u^{3} + 2 A_{r} u^{2} + A_{r}^{2} u - \chi = 0 \qquad \mathrm {at} \,\, u = u^{*}. \end{aligned}$$

To aid analysis, (25) is rewritten as a depressed cubic by making the change of variables $\hat{u} = u + 2A_{r}/3;$ then,

26 $$\begin{aligned} \hat{u}^{3} + p \hat{u} + q = 0 \qquad \mathrm {at} \;\; u = u^{*}, \end{aligned}$$

where

27 $$\begin{aligned} p = -\frac{A_{r}^{2}}{3},\quad q = -\frac{2}{27} A_{r}^{3} - \chi , \end{aligned}$$

where $\chi$ is as defined in (22). The three roots of the equation (26) may be succinctly expressed in the form

28 $$\begin{aligned} \hat{u}^{*}_{j} = 2\sqrt{\frac{-p}{3}}\cos {\left( \frac{1}{3}\arccos {\left( \frac{3 q}{2 p}\sqrt{\frac{3}{-p}}\right) } - \frac{2 \pi j}{3}\right) }, \quad j = 0,1,2, \end{aligned}$$

from which the optimal control $u^{*}$ may be constructed by inverting the change of variables,

29 $$\begin{aligned} u^{*} = \hat{u}^{*} - \frac{2}{3}A_{r}. \end{aligned}$$

For late-acting SIT the characterization is also defined by (29) with (27) and (28), but with $\chi$ now defined by (23).

The solutions (29) and (28) will either be all real, or be made up of one real root and two complex conjugate roots; on top of this, any real root may be negative. The character of each root—each value of *j* in (28)—is not fixed in time, and each may shift from real to complex as the parameters *p*, *q* evolve. When choosing the optimal solution, at each point in time, the non-zero, non-negative real root is selected. If (all) the real root(s) is (are) negative at a given time, the control is set to zero at that time. If all three roots are real and positive, the minimum value is taken. For $m_3 = 2,$ $\frac{\partial ^2 H}{\partial u ^2}>0$ is found so the solution is a minimum.

An analytical solution may also be found for a square root cost function, $m_3=\frac{1}{2}.$ In this case, for early-acting SIT, the solution is

30 $$\begin{aligned} \frac{\partial H}{\partial u} = \frac{1}{2}\theta _{3} u^{-\frac{1}{2}} k^{*}\mathrm {e}^{-\psi t} - \frac{\lambda _{1} \rho A^{2}}{A^{*}(A_r + u)^{2}} = 0 \qquad \mathrm {at} \,\, u = u^{*}. \end{aligned}$$

Making the change of variables $\mu = u^{\frac{1}{2}}$ (30) can be written as a depressed quartic equation for $\mu,$

31 $$\begin{aligned} \mu ^{4} + p \mu ^{2} + q \mu + r = 0, \end{aligned}$$

where

32 $$\begin{aligned} p = 2 A_r, \quad q = -2 A_{r}^{2}\chi , \quad r = A_{r}^{2}, \end{aligned}$$

where $\chi$ is as defined in (22). The solutions may then be written down using the canonical theory,

33 $$\begin{aligned} \mu = \pm _{1} \frac{1}{2}\sqrt{2 \bar{m}} \pm _{2} \sqrt{-\left( \frac{p + \bar{m}}{2} \pm _{1} \frac{\sqrt{2} q}{4 \sqrt{\bar{m}}}\right) }, \end{aligned}$$

where

34 $$\begin{aligned} \bar{m} = \frac{1}{2} \left( -\frac{2}{3} p + \frac{1}{3}\left( Q + \frac{\Delta _0}{Q} \right) \right) , \end{aligned}$$

with

35 $$\begin{aligned} Q = \left[ \frac{1}{2}\left( \Delta _1 + \sqrt{\Delta _1^{2} - 4 \Delta _0^{3}}\right) \right] ^{\frac{1}{3}}, \end{aligned}$$

and

36 $$\begin{aligned} \Delta _0 = p^2 + 12 r,\quad\Delta _1 = 2 p^3 + 27 q^2 - 72 p r. \end{aligned}$$

The characterization of the control may then be found by reversing the change of variables, $u^{*} = \mu ^{2}$. For late-acting SIT, the solution may also be expressed in the form (33), but with $\chi$ as defined in (23).

In the $m_{3} = \frac{1}{2}$ case, the concavity condition on $\partial ^2 H / \partial u^2$ holds only partially throughout the domain. It is found that

37 $$\begin{aligned} \frac{\partial ^2 H}{\partial u ^2} = \frac{2 \lambda _1 \rho A^2 A^{*^{2}}}{(A + u A^*)^3} - \frac{1}{4}\frac{\theta _3 k^*}{u^{\frac{3}{2}}}\mathrm {e}^{-\psi t}, \end{aligned}$$

which is positive for large enough *u*, but goes negative when

38 $$\begin{aligned} u^{\frac{3}{2}} \lesssim \frac{1}{8}\frac{\theta _3 A}{\lambda _1 \rho A^* N}\mathrm {e}^{-\psi t}. \end{aligned}$$

This relation generally corresponds to a very low value of control. Since small errors in a near-zero release ratio do not alter the overall cost or efficacy of the control strategy to an appreciable degree, this problem may be ignored. (A similar result is found in the case of late-acting SIT).

## Appendix B: $R_0$ and equilibria

### B.1 Basic reproductive ratio

For a constant mosquito population, the next generation matrix is given (using the $A = F - V,$ $G=FV^{-1}$ decomposition method of [58]) by

39 $$\begin{aligned} G = \left( \begin{matrix}0 &{} \frac{k^* b a}{\mu _A} \\ \frac{b c}{\gamma + w s} &{} 0 \end{matrix}\right) , \end{aligned}$$

for the rare initial infection scenario $(1-h)\sim 1,$ $(1-v)\sim 1.$ The basic reproductive ratio of disease (host to host) is given by the (square of the) dominant eigenvalue of *G*;

40 $$\begin{aligned} \begin{vmatrix} -\lambda&\frac{k^* b a}{\mu _A} \\ \frac{b c}{\gamma + w s}&-\lambda \end{vmatrix} = \lambda ^{2} - \frac{k^* b^2 a c}{\mu _A (\gamma + w s)} = 0, \end{aligned}$$

hence

41 $$\begin{aligned} R_0 = \frac{k^* b^2 a c}{\mu _A (\gamma + w s)}. \end{aligned}$$

The biting rate *b* appears as a square in the definition of $R_0,$ so *b* is considered to be an important parameter in determining the prevalence of disease.

From (41), the minimum number of vectors per host necessary to sustain the disease in a control-free environment is found to be

42 $$\begin{aligned} k_d = \frac{\gamma \mu _A}{b^2 a c} \approx 0.77, \end{aligned}$$

where default parameter values have been used (Table 1). Hence, even with less than one vector per host, the disease may be maintained at a low level.

### B.2 Equilibria and the effect of control

The population and endemic disease equilibria, in the absence of vector control, are found to be

43a $$\begin{aligned} L^* =&\, \frac{1}{\nu }\left[ \mathrm {e}^{m (\rho / 2 \mu _A - 1) - \mu _L} - 1 \right] ^{\frac{1}{\beta }}, \end{aligned}$$

43b $$\begin{aligned} A^* =&\, \frac{m L^*}{2 \mu _A}, \end{aligned}$$

43c $$\begin{aligned} h^* =&\, \frac{k^* b^2 c a - (\gamma + w s) \mu _A}{k^* b^2 c a + (\gamma + w s) b c}, \end{aligned}$$

43d $$\begin{aligned} v^* =&\, \frac{b c h^*}{b c h^* + \mu _A}. \end{aligned}$$

Vector control alters the equilibrium state of the system. Imagine a system in which disease has reached an endemic level [at the level given in (43c)] at some time $t<t_0,$ and vector control starts at $t = t_0,$ releasing *uA*(*t*) insects (rather than $u A^*$) for some constant release ratio *u*. The new equilibrium at which the system arrives at some time $t>t_0$ is then of key interest. For early-acting SIT, the new equilibria are

44 $$\begin{aligned} L^*_E =\, \frac{1}{\nu }\left[ \mathrm {exp}\left\{ \frac{1}{1+u} \frac{m \rho }{2 \mu _A} - m - \mu _L \right\} - 1 \right] ^{\frac{1}{\beta }},\quad A^*_E = \frac{m L^*_E}{2 \mu _A}, \end{aligned}$$

and (43c) and (43d); and for late-acting SIT the new equilibria are

45a $$\begin{aligned} L^*_L =\, \frac{1}{\nu }\left[ \mathrm {exp}\left\{ \frac{1}{1+u} \frac{m \rho }{2 \mu _A} - m - \mu _L \right\} - 1 \right] ^{\frac{1}{\beta }},&A^*_L =\, \frac{1}{1+u}\frac{m L^*_E}{2 \mu _A}, \end{aligned}$$

and (43c), (43d).

Critical release ratios $u = u_{\mathrm {c}}$ may be derived which cause disease fadeout ($h^*\rightarrow 0$); for early SIT, these release ratios are

46 $$\begin{aligned} u_{\mathrm {c}} = \frac{\rho m}{2 \mu _A}\left( m + \mu _L + \ln \left[ 1 + \left( 2 (\gamma + w s) \frac{\nu \mu _{A}^{2} N}{b^2 c a m}\right) ^{\beta }\right] \right) ^{-1} - 1, \end{aligned}$$

while for late SIT $u_{\mathrm {c}}$ satisfies

47 $$\begin{aligned} (1 + u_{\mathrm {c}})^{-\beta } \mathrm {exp}\left\{ \frac{1}{1+u_{\mathrm {c}}} \frac{m \rho }{2 \mu _A} - m - \mu _L \right\} = 1 + \left( 2 (\gamma + w s) \frac{\nu \mu _{A}^{2} N}{b^2 c a m}\right) ^{\beta }, \end{aligned}$$

where solutions of (47) may be found by Newton–Raphson iteration.

## Appendix C: A network model

The model outlined above may be extended to allow for movement of mosquitoes between population clusters. Each node (or population cluster) has its own set of state variables $L_i(t),$ $A_i(t),$ $h_i(t),$ $v_i(t)$ and controls $u_i(t),$ $w_i(t)$ satisfying the network model

48a $$\begin{aligned} \frac{\mathrm {d}L_i}{\mathrm {d}t} =&\, \rho A_i C(A_i, u_i) - f(L_i) L_i - (m + \mu _L) L_i ,\end{aligned}$$

48b $$\begin{aligned} \frac{\mathrm {d}A_i}{\mathrm {d}t} =&\, \frac{m}{2} L_i D(A_i, u_i) - \mu _A A_i - \frac{E_i A_i^{2}}{A_i^*} + \sum _{j\ne i}\sigma _{ij} \frac{E_j A_j^{2}}{A_j^*}, \end{aligned}$$

48c $$\begin{aligned} \frac{\mathrm {d}h_i}{\mathrm {d}t} =&\, \frac{A_i}{N_i} b a (1 - h_i) v_i - (\gamma + w_i s) h_i, \end{aligned}$$

48d $$\begin{aligned} \frac{\mathrm {d}v_i}{\mathrm {d}t} =&\, b c (1 - v_i) h_i - \left( \mu _A + \frac{1}{A_i}\frac{\mathrm {d}A_i}{\mathrm {d}t}\right) v_i - E_i v_i \frac{A_i v_i}{A_i^* v_i^*} + \frac{1}{A_{i}}\sum _{j\ne i}\sigma _{ij} E_j \frac{A_j^{2}v_j^{2}}{A_j^* v_j^*}, \end{aligned}$$

where

49 $$\begin{aligned} C(A_i, u_i) = \left\{ \begin{array}{ll} \displaystyle {\frac{A_i}{A_i + u_i A_i^{*}}, \quad \mathrm {early}\,\, \mathrm {SIT},} \\ \displaystyle {\quad \quad 1, \quad \quad \quad \, \mathrm {late}\,\, \mathrm {SIT},} \end{array} \right.&D(A_i, u_i) = \left\{ \begin{array}{ll} \displaystyle {\quad \quad 1, \quad \quad \quad \, \mathrm {early}\,\, \mathrm {SIT},} \\ \displaystyle {\frac{A_i}{A_i + u_i A_i^{*}}, \quad \mathrm {late}\,\, \mathrm {SIT}.} \end{array} \right. \end{aligned}$$

The tensor $\sigma$ defines the weighting of movement between nodes as a function of relative distance. An element $\sigma _{ij}$ gives the proportion of migrants from the *j*th population that settle in the *i*th. For the three-node case, this is defined as

50 $$\begin{aligned} \sigma = \left( \begin{matrix} 0 &{} \frac{\zeta (d_{12})}{D_2} &{} \frac{\zeta (d_{13})}{D_3} \\ \frac{\zeta (d_{21})}{D_1} &{} 0 &{} \frac{\zeta (d_{23})}{D_3} \\ \frac{\zeta (d_{31})}{D_1} &{} \frac{\zeta (d_{32})}{D_2} &{} 0 \end{matrix}\right) , \end{aligned}$$

where $d_{ij}$ is the distance between nodes *j* and *i* (thus $d_{ij} = d_{ji}$), the function $\zeta (d_{ij})$ is Laplace-type dispersal kernel,

51 $$\begin{aligned} \zeta (d_{ij}) = \frac{1}{2}\mathrm {e}^{-|d_{ij}|}, \end{aligned}$$

and $D_j = \sum _{k\ne j} \zeta (d_{kj}).$ In this way, $\sigma$ is normalised such that each column sums to unity, while each row does not. The parameter $E_i$ is a rate measuring the propensity for emigration of each population cluster. The parameters $A_i^*$ may be defined as the migration- and control-free equilibria, (43b).

### C.1 Optimal control of a network

A new per capita cost functional for a network of *n* identical population clusters is defined as,

52 $$\begin{aligned} J_{pc}[\varvec{x}(\varvec{u},\varvec{w})] = \int _0^T \mathrm {e}^{-\psi t} \sum _{i}^{n} \left[ \theta _{1} h_i^{m_{1}} + \theta _{2} h_i w_i^{m_{2}} + \theta _{3} k_i^{*} u_i^{m_{3}}\right] \mathrm {d}t, \end{aligned}$$

where $\varvec{u}$ and $\varvec{w}$ are vectors containing the controls $u_i$ and $w_i,$ respectively. The Hamiltonian becomes

53 $$\begin{aligned} H =&\,\, \mathrm {e}^{-\psi t} \sum _{i}^{n} \left[ \theta _{1} h_i^{m_{1}} + \theta _{2} h_i w_i^{m_{2}} + \theta _{3} k_i^{*} u_i^{m_{3}}\right] + \sum _{i}^{n}\left\{ \right. \lambda _{1}^{(i)}\left[ \rho A_i C(A_i, u_i) - (f(L_i) + m + \mu _L) L_i \right] \nonumber \\&\,+ \lambda _{2}^{(i)}\left[ \frac{m}{2} L_i D(A_i, u_i) - \mu _A A_i - \frac{E_i A_i^{2}}{A_i^*} + \sum _{j\ne i}\sigma _{ij} \frac{E_j A_j^{2}}{A_j^*}\right] \nonumber \\&+ \lambda _{3}^{(i)}\left[ \frac{A_i}{N_i} b a (1 - h_i) v_i - (\gamma + w_i s) h_i\right] \nonumber \\&\,+ \lambda _{4}^{(i)}\left[ b c (1 - v_i) h_i - \left( \mu _A + \frac{1}{A_i}\frac{\mathrm {d}A_i}{\mathrm {d}t} \right) v_i - E_i v_i \frac{A_i v_i}{A_i^* v_i^*} + \frac{1}{A_{i}}\sum _{j\ne i}\sigma _{ij} E_j \frac{A_j^{2}v_j^{2}}{A_j^* v_j^*} \right] \left. \right\} . \end{aligned}$$

The adjoint system for both the early- and late-acting SIT may be expressed, with the definitions of (49) in mind, as

54a $$\begin{aligned} \frac{\mathrm {d}\lambda _1^{(i)}}{\mathrm {d}t} =&\, -\frac{\partial H}{\partial L_i} = \lambda _{1}^{(i)}(f'(L_i)L_i + f(L_i) + m + \mu _{L}) - \frac{1}{2} \lambda _{2}^{(i)} m D(A_i, u_i) + \lambda _{4}^{(i)}\frac{m v_i}{2 A_i} D(A_i, u_i), \end{aligned}$$

54b $$\begin{aligned} \frac{\mathrm {d}\lambda _2^{(i)}}{\mathrm {d}t} =&\, -\frac{\partial H}{\partial A_i} = -\lambda _{1}^{(i)}\rho C(A_i, u_i) \left( 1 + A_i \frac{\partial }{\partial A_i}\log {C(A_i, u_i)}\right) - \lambda _{2}^{(i)}\bigg (\frac{m}{2} L_i \frac{\partial }{\partial A_i}D(A_i, u_i) - \mu _{A} \bigg ) \nonumber \\&\, -\lambda _{3}^{(i)}\frac{b a}{N_i}(1 - h_i) v_i - \lambda _{4}^{(i)} v_i \Bigg (\frac{m L_i}{2 A_i^{2}} D(A_i, u_i) \left[ 1 - A_i \frac{\partial }{\partial A_i}\log {D(A_i, u_i)} \right] - \frac{E_i v_i}{A_i^* v_i^*}\Bigg ) \nonumber \\&\, + \lambda _{4}^{(i)}\frac{1}{A_i^2}\sum _{j \ne i} \sigma _{ij} E_j \frac{A_j^{2}v_j^{2}}{A_j^* v_j^*} - 2 \frac{E_i A_i v_i^2}{A_i^* v_i^*} \sum _{j \ne i} \frac{\lambda _4^{(j)}}{A_j}\sigma _{ji} + 2 \frac{E_i A_i}{A_i^*}\left( \lambda _2^{(i)} - \sum _{j \ne i} \lambda _2^{(j)} \sigma _{ji} \right) , \end{aligned}$$

54c $$\begin{aligned} \frac{\mathrm {d}\lambda _3^{(i)}}{\mathrm {d}t} =&\, -\frac{\partial H}{\partial h_i} = -\mathrm {e}^{-\psi t}(m_{1} \theta _{1} h_i^{m_{1}-1} + \theta _{2} w_i^{m_{2}}) + \lambda _{3}^{(i)}\left( \frac{A_i}{N_i} b a v_i + \gamma + w_i s\right) - \lambda _{4}^{(i)} b c (1 - v_i), \end{aligned}$$

54d $$\begin{aligned} \frac{\mathrm {d}\lambda _4^{(i)}}{\mathrm {d}t} =&\, -\frac{\partial H}{\partial v_i} = -\lambda _{3}^{(i)}\frac{A_i}{N_i} b a (1 - h_i) + \lambda _{4}^{(i)}\left( b c h_i + \mu _A + \frac{1}{A_i}\frac{\mathrm {d}A_i}{\mathrm {d}t}\right) \nonumber \\&+ 2 E_i \frac{A_i^2 v_i}{A_i^* v_i^*}\left( \frac{\lambda _4^{(i)}}{A_i} - \sum _{j \ne i} \frac{\lambda _4^{(j)}}{A_j} \sigma _{ji}\right) , \end{aligned}$$

where $1 - \sum _{j\ne i}\sigma _{ji} = 0$ has been used in (54b).

The optimality conditions that define the best-practice for using artemisinin and vector-release controls are

55 $$\begin{aligned} \frac{\partial H}{\partial u_i} = 0 \quad \mathrm {at} \,\, u_i = u_i^{*},\quad \frac{\partial H}{\partial w_i} = 0 \quad \mathrm {at} \,\, w_i = w_i^{*}. \end{aligned}$$

The controls $u_i$ and $w_i$ never appear in equations $\mathcal {L}_j \mathbf{x}_j,$ for nodes *i*, *j*, differential operator $\mathcal {L}$ and vector of states and adjoints $\mathbf{x}$—in either (48) or (54)—where $j \ne i,$ hence the results of the previous section are still valid for the network model. Then, the optimal controls for each pricing structure and each population cluster may be defined as in (18), (21) and (29).
